# Electron Diffraction Tomography on Two-Phase Nanolamellae of Topochemically Synthesized Cu(Sb_2_S_3_)Cl

**DOI:** 10.1021/acs.inorgchem.4c01674

**Published:** 2024-06-08

**Authors:** Wilder Carrillo-Cabrera, Oliver Dreimann, Matthias A. Grasser, Prosun Santra, Silvan Kretschmer, Arkady V. Krasheninnikov, Michael Ruck

**Affiliations:** † Faculty of Chemistry and Food Chemistry, Technische Universität Dresden, 01062 Dresden, Germany; ‡ Max Planck Institut für Chemische Physik fester Stoffe, 01187 Dresden, Germany; § Helmholtz-Zentrum Dresden-Rossendorf, 01328 Dresden, Germany

## Abstract

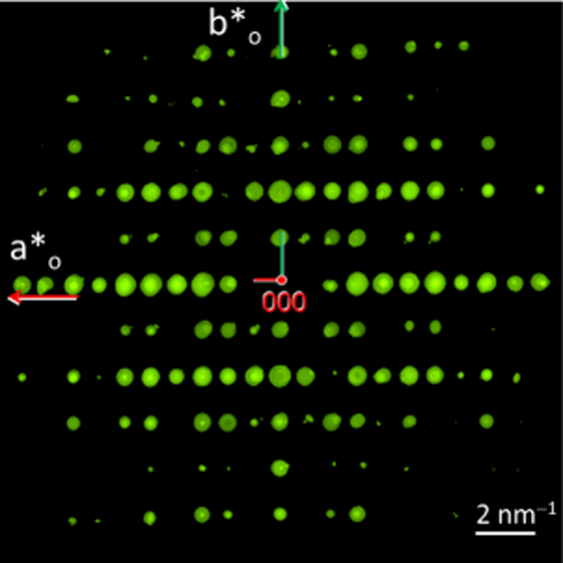

The dark red semiconductor Cu(Sb_2_S_3_)Cl was obtained by leaching the layered precursor Cu(Sb_2_S_3_)[AlCl_4_] in a 0.1 M aqueous HCl solution. The selective extraction of AlCl_3_ yielded a mica-like lamellar product of poor crystallinity. Misalignment of lamellae down to the nanoscale prevented structure determination by conventional single-crystal X-ray diffraction, but a combination of transmission electron microscopy, selected area electron diffraction, and selected area electron precession diffraction tomography on a nanoscale spot with largely ordered crystalline lamellae revealed the crystal structures of two intergrown modifications. Orthorhombic o-Cu(Sb_2_S_3_)Cl and monoclinic m-Cu(Sb_2_S_3_)Cl have similar layers to the precursor and differ only in the stacking of the layers. These consist of uncharged Sb_2_S_3_ strands, whose sulfide ions, together with chloride ions, coordinate the copper(I) cations. Only one chloride ion remained from the [AlCl_4_]^−^ group. DFT calculations confirm the structure solution for the orthorhombic form and suggest that the monoclinic structure is metastable against transformation to o-Cu(Sb_2_S_3_)Cl.

## Abstract 2

The new layered semiconductor Cu(Sb_2_S_3_)Cl can only be obtained by leaching of AlCl_3_ from the precursor compound Cu(Sb_2_S_3_)[AlCl_4_]. The topochemical reaction results in poorly crystalline flakes. Manual three-dimensional electron diffraction tomography on the nanoscale allowed to determine the crystal structures of two intergrown and twinned polytypes. Quantum chemical calculations reveal that one of them is stable, while the other is metastable.

## Introduction

Detailed knowledge of the crystal structure of nanomaterials, be it particles, thin layers, or domains, is essential for understanding their properties. However, many of the common diffraction methods reach their limits when the underlying structures are complex. Even high-resolution transmission electron microscopy (HRTEM) has problems when there is no simple periodicity in the viewing direction. Based on great scientific work from the last three decades, three-dimensional electron diffraction for structure determination has developed into a method that can no longer be mastered only by experts with highly specialized skills and equipment. A decisive step was the introduction of precession electron diffraction tomography (PEDT), with which significantly more reflections can be measured than in a static diffraction experiment.^1−10^ Electron diffraction images are typically recorded by tilting the crystal in 1° increments around the tilt axis of the transmission electron microscope (TEM) goniometer and using a precessed beam (precession angle of ca. 1°) for each tilt.^11^ The images from this incremental rotation crystal measurement are then combined by software to form a 3D diffraction image, and the reflection intensities are integrated. The precession of the beam reduces dynamic scattering effects, which are difficult to model. Therefore, the diffraction intensities can be directly used for structure solution and refinement using programs developed for X-ray diffraction data.^12,13^ Since electrons interact much more strongly with matter than X-rays, the PEDT method can be applied successfully for very small scattering volumes, such as nanoparticles or nanoscopic domains of crystals. Modern dedicated electron diffractometers are commercially available; however, manual PEDT on a conventional TEM is a more cost-effective but a more time-consuming method of acquiring 3D ED data.

Based on previous experience with structure solution from electron diffraction or PEDT data,^14−17^ we tested the method on the product of an unusual topochemical reaction. This continues our investigations on the synthesis of new compounds by heterogeneous reactions including mass transport and structural transformations in the solid state.^18−22^ The compounds obtained in this way are often, but not necessarily, metastable and cannot be produced by other methods. Yet, the applied chemical modifications can for example change the magnetism and induce superconductivity^23^ or create an excellent oxide ionic conductor.^24^

**Figure 1:**
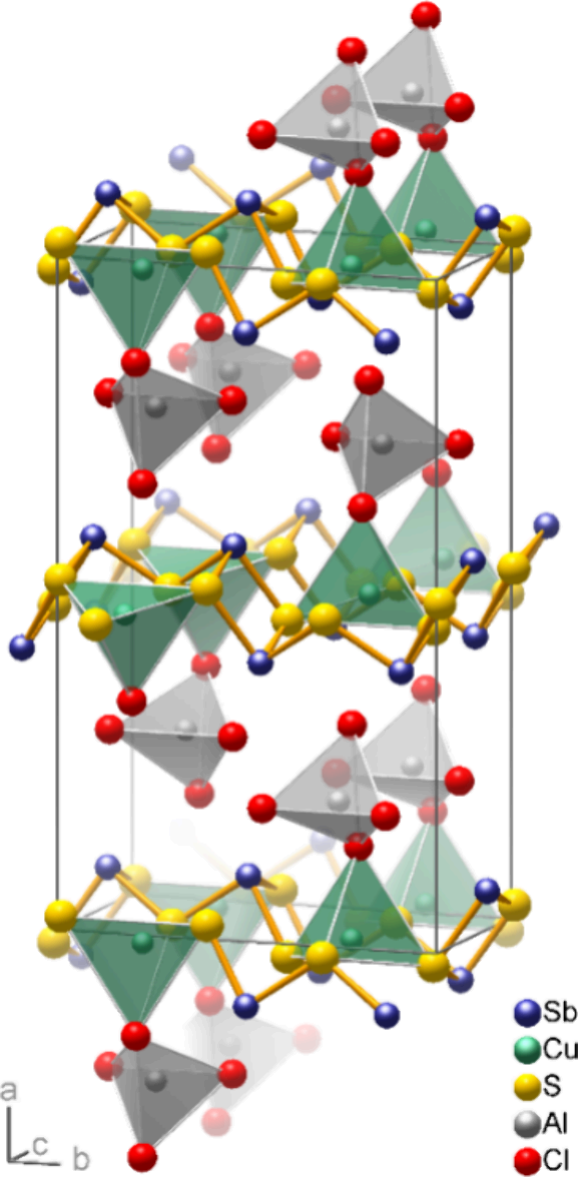
Crystal structure of precursor compound Cu(Sb_2_S_3_)[AlCl_4_].

In the underlying case, the starting material was Cu(Sb_2_S_3_)[AlCl_4_], a recently discovered semiconducting compound, which we obtained by reacting Sb_2_S_3_ and CuCl in an ionic liquid at 200 °C.^25^ The orthorhombic layered structure comprises uncharged Sb_2_S_3_ strands whose sulfide ions coordinate the copper(I) cations together with chloride ions of the [AlCl_4_]^−^ tetrahedra (Figure 1 and Figure S1 of the Supporting Information). The optical band gap of about 2.1 eV is in the range reported for amorphous Sb_2_S_3_ (metastibnite, 1.7 to 2.8 eV)^26−28^ and suitable for optical emitters and sensors.^29^ Unfortunately, the compound is very sensitive to moisture.

We discovered that when the compound is treated in a 0.1 M aqueous HCl solution, the crystals are transformed into an aluminum-free, water stable, dark red to brownish material.^25^ The crystals are largely preserved, but their crystallinity suffers strongly. A topochemical reaction seems likely since the two in-plane lattice parameters of the product (∼10.7 and ∼5.6 Å) are similar to those of the precursor. The chemical composition of the product is close to “CuSb_2_S_3_Cl” according to energy-dispersive X-ray (EDX) spectroscopy. It appeared that AlCl_3_ was quantitatively leached from Cu(Sb_2_S_3_)[AlCl_4_] while the remaining layers were preserved.^25^ With respect to the acidic solution and the locally probably even lower pH (formation of hydrochloric acid), this is an unexpected reaction product. At higher pH, only copper sulfide or antimony oxide chlorides are obtained.

Unfortunately, neither the crystal structure nor even the lattice periodicity in the stacking direction could be determined by means of laboratory single-crystal X-ray diffraction. In the electron microscope, the reaction product turned out to be polycrystalline with mica-like lamellar crystallites. To obtain the missing information, a combined TEM, selected-area electron diffraction (SAED), and selected-area precession electron diffraction tomography (SA-PEDT) study was started. Two crystal structures of topochemically synthesized Cu(Sb_2_S_3_)Cl were solved ab initio using 3D PEDT data of nanoscopic crystalline spots of the heavily disordered and intergrown particles.

## Results and Discussion

### Microstructure and Chemical Analysis

**Figure 2:**
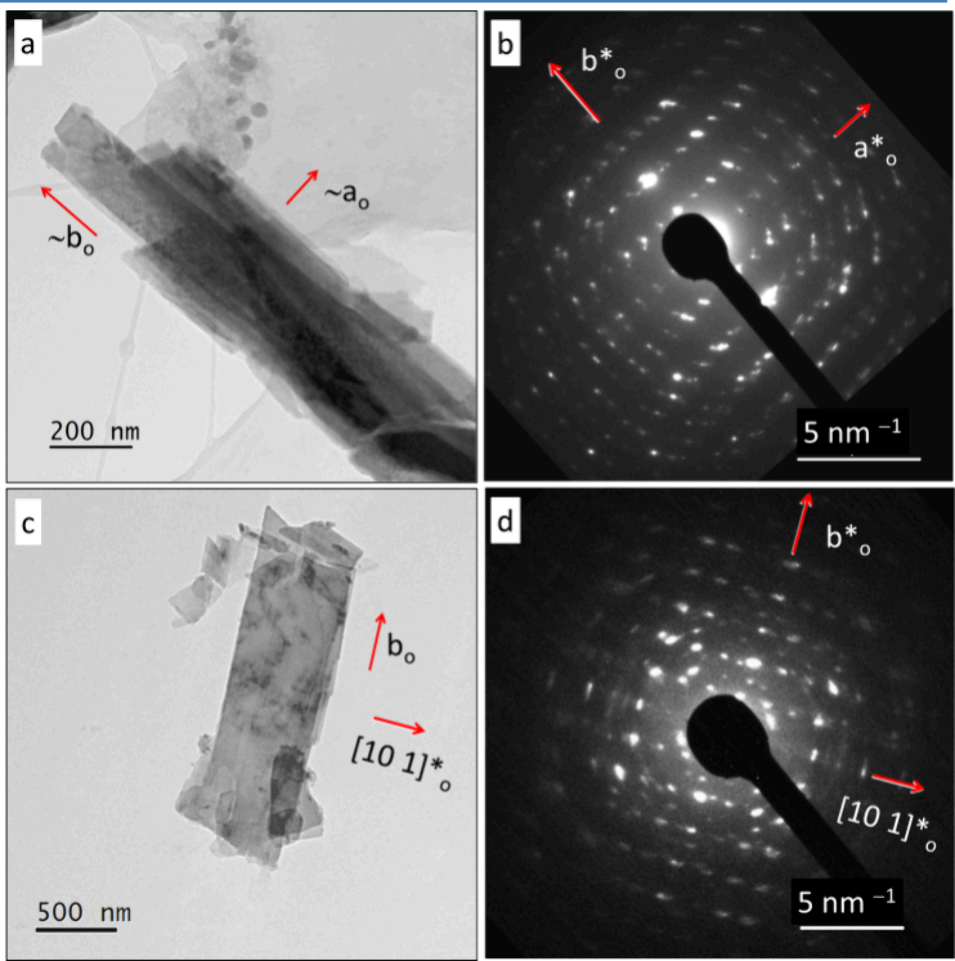
Sonicated Cu(Sb_2_S_3_)Cl particles observed by TEM. Directions were assigned based on an orthorhombic lattice. (a, c) Images of aggregates of lamellar crystals of Cu(Sb_2_S_3_)Cl with (b, d) respective electron diffraction patterns. The lamellae are attached to each other in nearly the same orientation.

Figure 2 shows TEM images and SAED patterns of two examples of thin platelets obtained by treating Cu(Sb_2_S_3_)[AlCl_4_] crystals for 5 min with a 0.1 m aqueous HCl solution under argon flow followed by brief sonication in ethanol. The size of the particles is in the range of a few microns and, since no surfactant has been used, they have a tendency to agglomerate. Even the thinner platelets consist of stacks of bent or twisted lamellae that are aligned nearly in the same direction (slightly rotated from each other). Most of them lie on the supporting carbon film with their main face perpendicular to the electron beam.

Under the electron beam in high vacuum, only platelets thicker than ca. 20 nm are stable for several hours. Thinner lamellae become porous and almost amorphous after about 30 min under the electron beam. A few platelets were found to be tilted to the electron beam, revealing their *c* axis. They became amorphous within a few seconds of electron beam exposure. This instability prevented HRTEM investigations of the stacking sequence and intergrowth structure.

EDX analysis on two isolated particles selected for SAED resulted in compositions Cu_1.0_Sb_2_S_2.6_Cl_0.9_ and Cu_1.0_Sb_2_S_2.5_Cl_0.9_, in agreement with previous experiments. Remarkably, no aluminum residues were detected. Even if the standard-less EDX analysis in the TEM is not as precise, the composition found was important for the success of the ab initio crystal-structure solution.

### Crystal Structure Determination

To choose crystals suitable for selected-area precession electron diffraction tomography (SA-PEDT), several crystals from two different batches were studied by TEM. Almost all crystals had their [001] axis parallel to the electron beam (vertical) due to the platelet habitus with the main face (001), and because of the mentioned stability issues under the electron beam, only those could be investigated.

**Figure 3:**
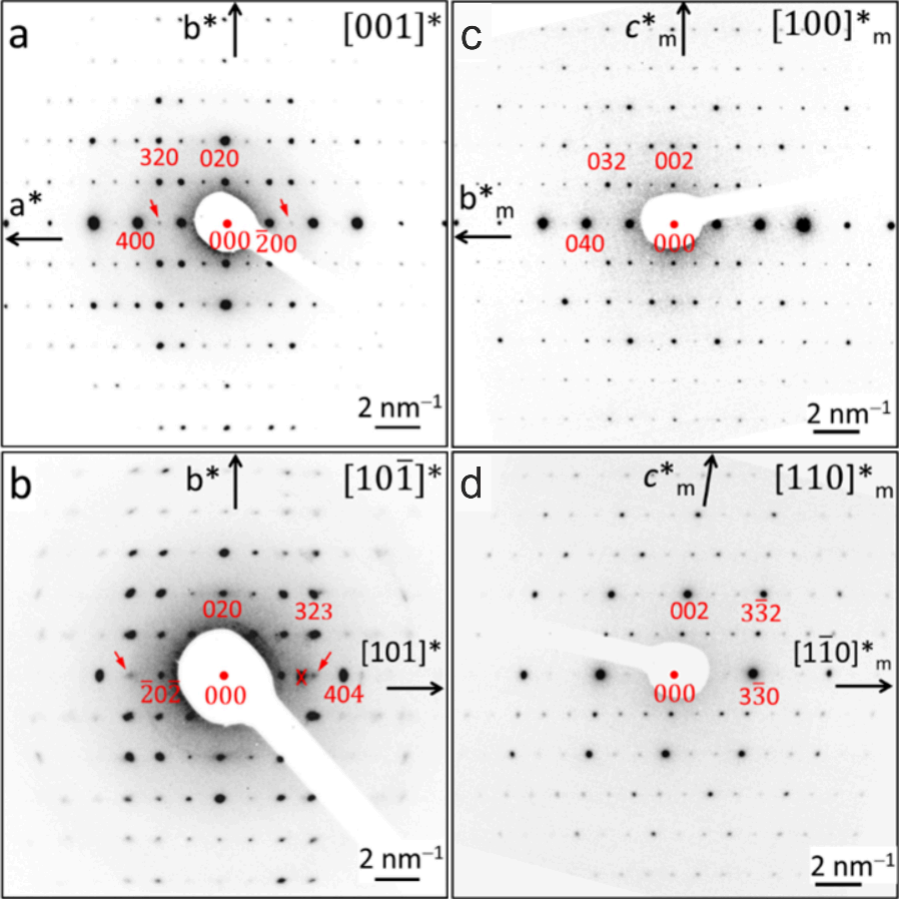
SAED electron diffraction images. Left: crystal 1o with 90% o-Cu(Sb_2_S_3_)Cl (a) along the [001]*_o_ zone axis and (b) along the [101̅]*_o_ zone axis. Some weak reflections (red arrows) might have zero intensity under kinematic conditions. In panel (b), a reflection originated by a satellite crystal is marked by a red cross. Right: crystal 3m with 63% m-Cu(Sb_2_S_3_)Cl (c) along the [100]*m zone axis and (d) along the [110]*m zone axis.

The inspection of the SAED patterns of various platelets revealed two different lattices (Figure 3). The final assignment orthorhombic (o) or monoclinic (m) was done after the crystallographic analysis of the 3D electron diffraction data. Two predominantly orthorhombic domains (crystal 1o and 2o, Figure S3) and one predominantly monoclinic domain (crystal 3m, Figure S6) were chosen. The orthorhombic structure appeared to be primitive, but a space group could not be assigned reliably because some weak reflections might have zero intensity under kinematic conditions. For the mostly monoclinic crystal, the lattice seemed to be primitive and the space group *P*2_1_/*m* appeared to be likely. All three crystals (1o, 2o, and 3m) were used for the acquisition of the 3D diffraction patterns using SA-PEDT. After crystal structure determination and refinement, it was found that the orthorhombic and monoclinic crystals contain coherent intergrowth defects of each other phase.

In the SA-PEDT experiment, an electron microscope was used as a one-circle electron diffractometer in an incremental (manual) rotation mode. The collected 2D-PED data were used for reconstruction of the diffraction volume. No correction was applied to the electron intensity data. Details are described in the Experimental Section. The same orthorhombic primitive unit cell was identified for crystal 1o and crystal 2o. For crystal 3m, a monoclinic primitive lattice was found. Since the 2D electron diffraction images were not calibrated accurately for the actual microscope conditions, lattice parameters determined by powder X-ray diffraction were used. The crystal structures of o- and m-Cu(Sb_2_S_3_)Cl were solved based on the three sets of integrated SA-PEDT data using the charge-flipping algorithm.^30,31^ All three investigated crystal lamellae consisted predominantly of one modification but comprised also fractions of the other form. Moreover, the monoclinic domains were twinned. Despite these unfavorable boundary conditions and without additional information except the elements included (from EDX analysis) the structure solutions succeeded. The crystallographic data are summarized in the Supporting Information (Tables S1 to S3). Selected interatomic distances are listed in Table 1.^32^

**Table 1:** Selected Interatomic Distances (Å) for Both Modifications of Cu(Sb_2_S_3_)Cl and for o-Cu(Sb_2_S_3_)Cl after DFT-Based Optimization (Averaged in Space Group Symmetry)^a^

atom pair		crystal 1o	crystal 2o	DFT	atom pair		crystal 3m
Cu1–	S1	2.31(3)	2.50(2)	2.28	Cu1–	S1	2.38(5)
	S3 [2×]	2.50(3)	2.45(2)	2.29		S2 [2×]	2.29(3)
	Cl1	2.48(5)	2.31(5)	2.39		Cl1	2.34(6)
Cu2–	S4 [2×]	2.24(2)	2.25(2)	2.27	Cu2–	S4 [2×]	2.31(4)
	S2	2.37(3)	2.28(3)	2.28		S3	2.31(7)
	Cl2	2.43(5)	2.61(5)	2.50		Cl2	2.47(11)
Sb1–	S2	2.30(2)	2.30(2)	2.49	Sb1–	S1	2.49(3)
	S4	2.61(2)	2.62(2)	2.54		S2	2.50(3)
	S3	2.87(3)	2.72(3)	2.54		S2	2.72(3)
Sb2–	S4	2.40(3)	2.56(3)	2.49	Sb2–	S4	2.38(5)
	S1	2.49(3)	2.59(2)	2.49		S3	2.29(3)
	S3	2.51(2)	2.39(2)	2.53		S4	2.34(6)

a Note that the symmetry-independent atoms in the two polytypes are different.

### Crystal Structure of o-Cu(Sb_2_S_3_)Cl

**Figure 4:**
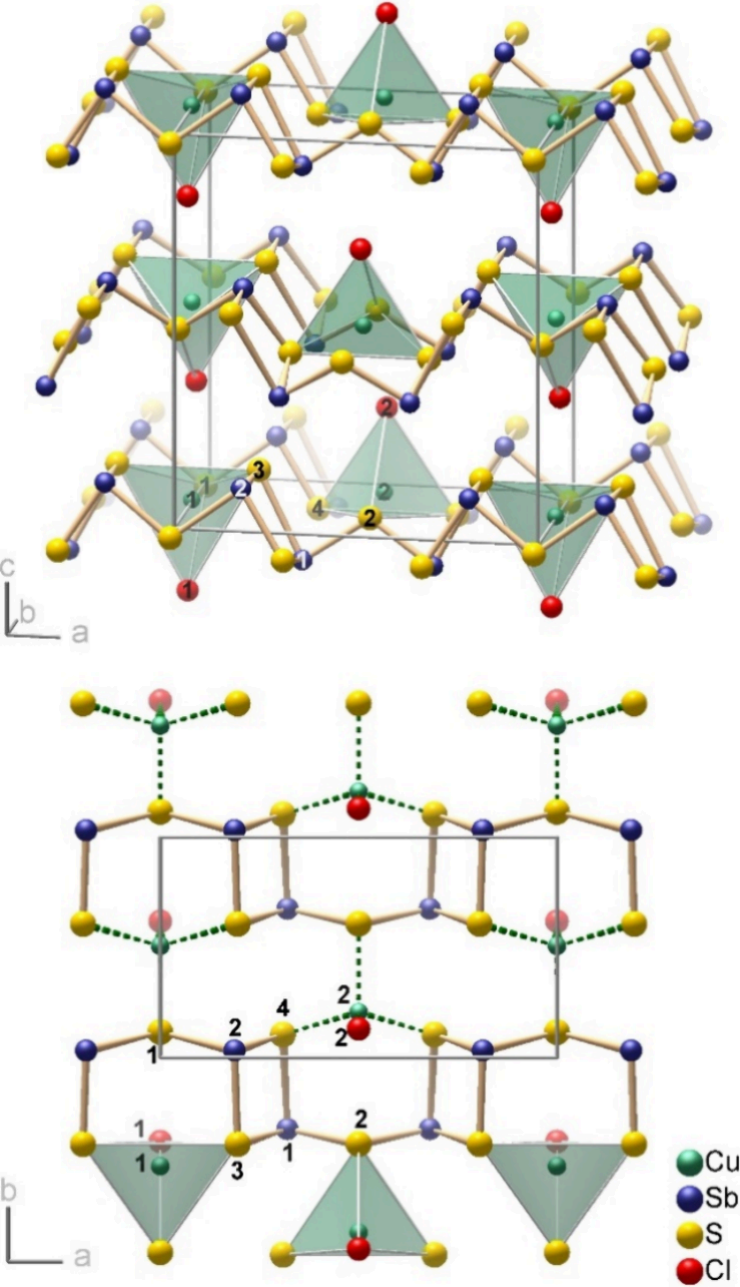
Top: crystal structure of o-Cu(Sb_2_S_3_)Cl. The coordination polyhedra of copper(I) cations are emphasized. Bottom: top view of a single _∞_^2^[Cu(Sb_2_S_3_)Cl] layer.

The crystal structure in the space group *Pmc*2_1_ represents a new structure type (*oP*28) and consists of _∞_^2^[Cu(Sb_2_S_3_)Cl] layers, which are stacked along the *c*-axis (Figure 4). _∞_^1^[Sb_2_S_3_] strands with three-bonded antimony and two-bonded sulfur atoms run parallel to the *a*-axis. The copper(I) cations are in distorted tetrahedral coordination by three sulfur atoms and a chloride ion. At room temperature, the experimentally determined lattice parameters are *a*_o_ = 10.617(1) Å, *b*_o_ = 5.898(1) Å, and *c*_o_ = 11.730(8) Å. For comparison, those of the orthorhombic precursor (space group *Pnma*) are *a*_p_ = 18.341(3) Å, *b*_p_ = 10.810(2) Å, and *c*_p_ = 5.799(1) Å (*a*_o_ ≈ *b*_p_, *b*_o_ ≈ *c*_p_). The volumes differ by 104 Å^3^ per formula unit. The base area of the unit cell parallel to the layers is almost identical in both compounds, so that the volume change arises mainly from a decrease of the lattice parameter in the stacking direction. The removal of one equivalent AlCl_3_ per formula unit has only a slight impact on the structure of the individual _∞_^2^[Cu(Sb_2_S_3_)Cl] layer. To delete the respective atoms from the structure model of the precursor is a rather good approximation. While the interatomic distances in the layer (Table 1) do not change much, the strongest effect is seen in the bond angles at the Sb atoms. The shortest interlayer distance (Sb1···Cl2, 3.11 Å) is similar to that in Cu(Sb_2_S_3_)[AlCl_4_] (Sb···Cl1, 3.20 Å). The accuracy of the structure determinations based on SA-PEDT data is not high enough for a meaningful comparison in greater detail. This becomes particularly clear from the fact that some atomic coordinates of the crystals 1o and 2o deviate from each other by more than three standard deviations. The samples are definitely at the limit of what can be described as crystal.

Therefore, we performed a density functional theory (DFT)-based optimization of the total energy by variation of the lattice parameters and atomic positions without applying any point group symmetry. The thereof obtained atomic positions (Table S4) are very close to the symmetry of the space group *Pmc*2_1_ and reproduce the experimentally determined structure model (Table S2, Figure S10). The calculated lattice parameters are *a*_oc_ = 10.618 Å, *b*_oc_ = 5.8958 Å, and *c*_oc_ = 11.115 Å. While the experimental and calculated values for *a*_o_ and *b*_o_ are almost identical, there is a considerable difference in *c*_o_ – *c*_oc_ of 0.615 Å. Since *c*_o_ is the stacking direction, the main differences between the structure models lie in the interlayer distance. This may be due to several reasons, such as uncertainties in the evaluation of the diffraction experiment, some residues from the leaching process, or shortcomings of the DFT in calculating weak interactions over long interatomic distances. The calculated bond lengths are closer to those obtained from the diffraction data of crystal 2o. About half of them match within three standard deviations, while the rest differ by up to 12%. Moreover, the DFT-generated model has a narrower range of bond length, which was also found in the precursor Cu(Sb_2_S_3_)[AlCl_4_] (Cu–S 2.30 ± 0.01 Å; Cu–Cl 2.51 Å; Sb–S 2.48 ± 0.01 Å; Sb–Cl 3.20 Å).

In the precursor and o-Cu(Sb_2_S_3_)Cl, the layer group symmetry is the same, *p* (1) 2_1_*/m* 1 and *p* 2_1_*/m* 1 (1), with respect to the different unit cell settings. However, the densification along the stacking direction results in a lateral shift of about 1.2 Å along the shortest axis between neighboring layers, which also has consequences for the space group symmetry. Different from Cu(Sb_2_S_3_)[AlCl_4_] (*Pnma*), the structure of o-Cu(Sb_2_S_3_)Cl is not centrosymmetric (*Pmc*2_1_). In the latter, the screw axes and the centers of inversion that are intrinsic to the single layer do not apply to the entire structure (pseudosymmetry). Formally, there is a group-subgroup relationship between the two structures, but the mentioned shift between neighboring layers creates substantial differences between the derived and actual atomic coordinates of o-Cu(Sb_2_S_3_)Cl.

### Crystal Structure of m-Cu(Sb_2_S_3_)Cl

**Figure 5:**
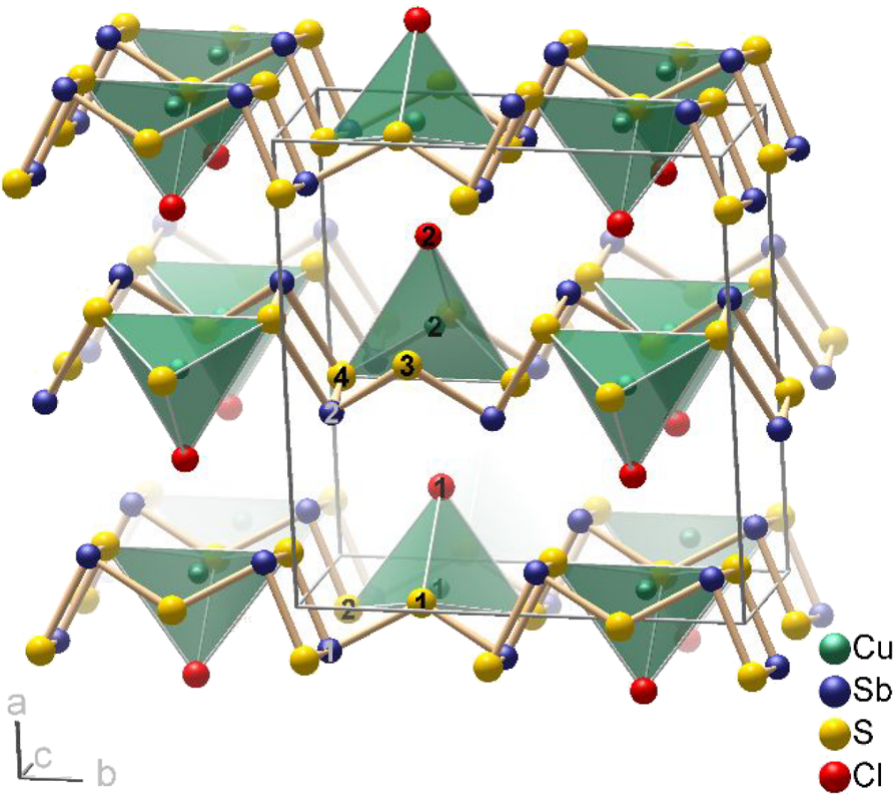
Crystal structure of m-Cu(Sb_2_S_3_)Cl. The coordination polyhedra of the copper(I) cations are emphasized. For representations of the _∞_^2^[Cu(Sb_2_S_3_)Cl] layers around *x* = 0 and *x* = 0.5, see Figure S8.

The monoclinic form consists of the same _∞_^2^[Cu(Sb_2_S_3_)Cl] layers but is a different polytype. The lattice parameters at room temperature are *a*_m_ = 12.219(8) Å, *b*_m_ = 10.617(1) Å, *c*_m_ = 5.884(1) Å, and β_m_ = 105.17(3)°. The unit cell volume is only 0.2% larger than that for the orthorhombic form. Again, the layer group symmetry *p* (1) 2_1_*/m* 1 is preserved for both crystallographically independent layers, and the space group *P*2_1_*/m* is formally a maximum subgroup of the precursor’s space group *Pnma*. Analogous to Cu(Sb_2_S_3_)[AlCl_4_], the layers lie parallel to the (100) plane, and the *a*-axis is the stacking direction (Figure 5).

Projected along the *c*-axis (i.e., the shortest axis), the structures of m-Cu(Sb_2_S_3_)Cl and Cu(Sb_2_S_3_)[AlCl_4_] look very similar. However, in relation to the precursor’s structure, the layers are shifted against each other by Δ*z* ≈ 1/4 following the monoclinic angle and destroying the glide planes of *Pnma*. While in m-Cu(Sb_2_S_3_)Cl all shift vectors Δ*z* have the same orientation, in o-Cu(Sb_2_S_3_)Cl, they point alternately in opposite directions (Figure S10). Due to these substantial shifts, it makes no sense to extend the group-subgroup relationships to the atomic positions. DFT-based optimizations of the m-Cu(Sb_2_S_3_)Cl structure converged in the structure of o-Cu(Sb_2_S_3_)Cl (Figure S12), suggesting that the monoclinic form observed in the experiments is likely metastable.

**Figure 6:**
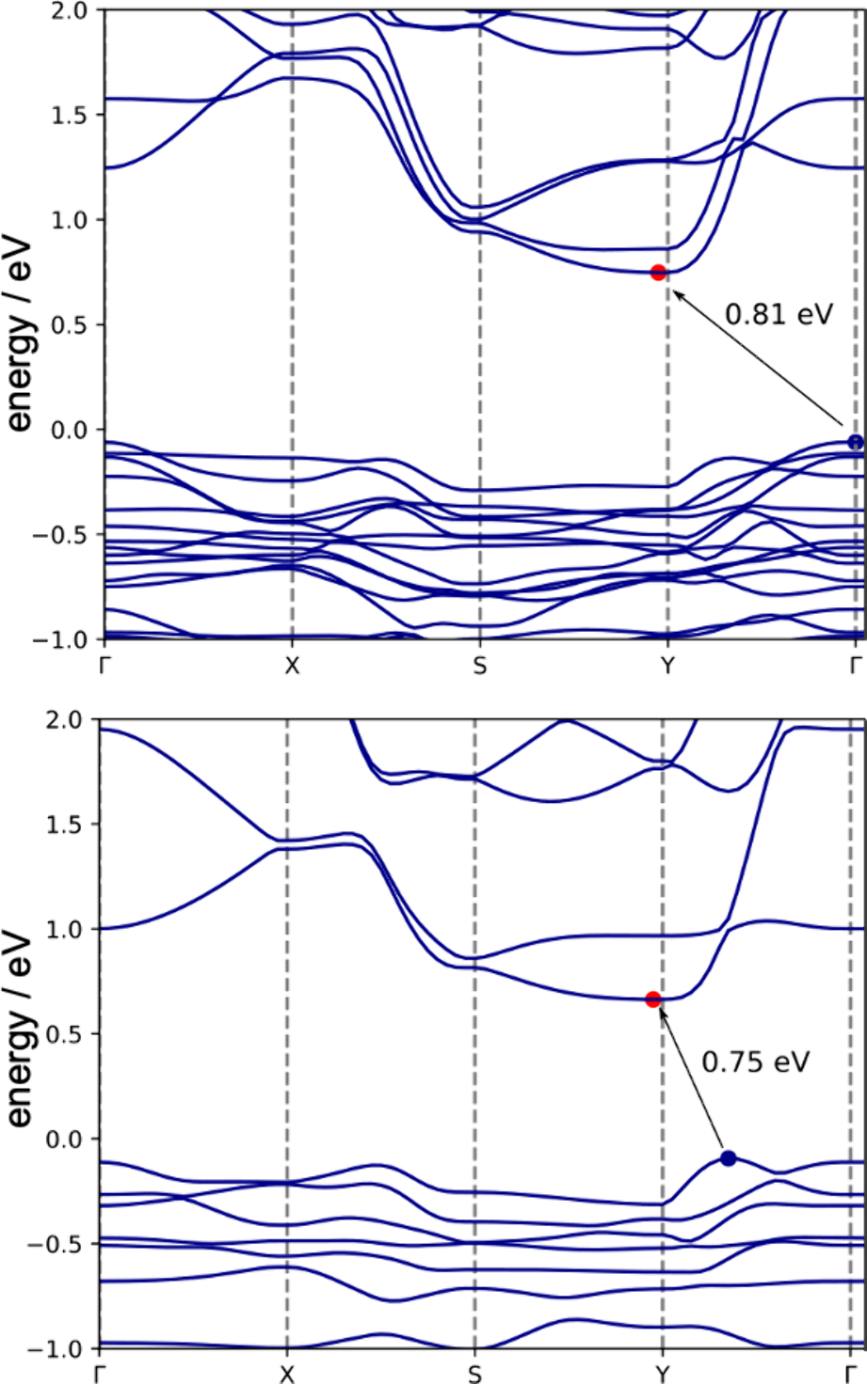
Electronic band structure (PBE functional) of bulk o-Cu(Sb_2_S_3_)Cl (top) and a single _∞_^2^[Cu(Sb_2_S_3_)Cl] layer (bottom).

Electronic structure of o-Cu(Sb_2_S_3_)Cl: Calculations were performed within the framework of density functional theory (DFT) using the plane-wave projector augmented-wave (PAW) method along with the Perdew–Burke–Ernzerhof (PBE) exchange-correlation functional.^33,34^ Van der Waals interactions were taken into account using the many-body dispersion method.^35,36^ The calculated band structures for bulk o-Cu(Sb_2_S_3_)Cl and a single _∞_^2^[Cu(Sb_2_S_3_)Cl] layer are shown in Figure 6. For the crystal, an indirect band gap of 0.8 eV was found. The single sheet has a slightly smaller band gap of 0.75 eV. The gap is also indirect, but the valence band maximum is shifted away from the Γ point. As DFT with local or semilocal functionals underestimates the gap, we also carried out G0W0 calculations for the bulk, which gave a quasiparticle gap of 1.6 eV. This corresponds with the dark red color of the compound and is close to the (indirect) band gap of 1.54 eV derived from a UV spectrum (Figure S11).

The analysis of the local density of states (Figure S13) indicates that 3d-states of Cu atoms mostly contribute to the valence band maximum, while electronic states of all atoms contribute to the conduction band minimum. The Bader charges were found to be Cu^0.5+^, Sb^1.2+^, S^0.68–^, and Cl^0.64–^, indicating strongly heteropolar bonding. The substantial dispersion of the bands above the Fermi level, which have strong contributions of Sb and S, shows that the bonding in the Sb_2_S_3_ strands is nonetheless covalent.

## Conclusions

Cu(Sb_2_S_3_)Cl, the first compound reported in this quaternary system, was obtained by the acidic leaching of Cu(Sb_2_S_3_)[AlCl_4_]. Reaction, mass loss, and associated shrinkage break the crystals of Cu(Sb_2_S_3_)[AlCl_4_] into intergrown nanoplatelets with very poor crystallinity. Electron crystallography on nanoscopic spots with largely ordered crystalline lamellae allowed us to determine the crystal structures of two intergrown and twinned polytypes of Cu(Sb_2_S_3_)Cl without additional information beyond the included elements (obtained from EDX analysis). The layered crystal structures of both modifications are closely related to the structure of the precursor, given that one equivalent of AlCl_3_ per formula unit was extracted. The leaching can undoubtedly be classified as a topochemical reaction. DFT calculations revealed that the monoclinic polymorph of Cu(Sb_2_S_3_)Cl is likely metastable, while the orthorhombic structure is stable and correct within the limits of the methods applied. It is all the more remarkable that attempts to synthesize Cu(Sb_2_S_3_)Cl directly from CuCl and Sb_2_S_3_ have not been successful. Moreover, Cu(Sb_2_O_3_)Cl and Cu(Sb_2_O_3_)Br have different structures consisting of Sb_4_O_6_ cages and CuCl or CuBr layers.^37^ Thus, the use of the structurally close precursor and the moderate conditions of the topochemical reaction seem to be indispensable for the synthesis of Cu(Sb_2_S_3_)Cl.

## Experimental Section

### Synthesis of the Precursor Cu(Sb_2_S_3_)[AlCl_4_]

According to the literature,^25^ Cu(Sb_2_S_3_)[AlCl_4_] is obtained by heating a mixture of CuCl and Sb_2_S_3_ (molar ratio of 2:1) in the ionic liquid [BMIm]Cl·4.4AlCl3 to 200 °C followed by cooling down to room temperature. The IL acts both as a solvent and reactant.
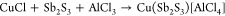


For a better understanding of the formation of Cu(Sb_2_S_3_)[AlCl_4_], differential scanning calorimetry (DSC) was performed. In a fused, evacuated silica ampule, the reaction mixture was heated to 250 °C at a constant rate of 2 K min^–1^. The DSC data (Figure S2) showed an endothermic effect at 158 °C, which we attribute to the dissolution of the starting materials in the ionic liquid. The two exothermic effects at 164 and 180 °C are associated with chemical reactions. During the subsequent cooling at the same rate, the crystallization of the target compound is indicated by a broad exothermic effect that has its onset at about 130 °C. Based on these data, an optimized protocol was developed.

All compounds were handled in an argon-filled glovebox (MBraun; *p*(O_2_)/*p*^0^ < 1 ppm, *p*(H_2_O)/*p*^0^ < 1 ppm). The reactions were carried out in silica ampules with a length of 120 mm and diameter of 14 mm. The ampule was loaded with a mixture of 67.9 mg of Sb_2_S_3_ (0.2 mmol, Chempur, min. 98%), 39.6 mg of CuCl (0.4 mmol, Sigma-Aldrich, 99%), and 300.0 mg of AlCl_3_ (2.25 mmol, sublimed three times), which were ground together in an agate mortar. Afterward, 90 mg of [BMIm]Cl (0.52 mmol, 99%, Iolitec, dried under vacuum at 100 °C) was added, which induces liquefaction of the mixture.

The evacuated and sealed ampule was shaken with a vortex mixer before it was placed in a tubular furnace. It was quickly heated to 165 °C at 2 K min^–1^ and kept at this temperature for 1 day to ensure complete dissolution of the starting materials. Then, the ampule was cooled down to 130 °C at a rate of −5 K h^–1^ and to 123 °C at −1 K h^–1^. After 5 days, the ampule was slightly tilted to separate the orange-red crystals from the liquid reaction medium and then cooled to room temperature at −1 K h^–1^. After opening the ampule, the IL was decanted from the crystals. The crystals were washed with dichloromethane (Fisher Scientific, HPLC grade, amylene stabilized) that had been dried and degassed (MBraun Solvent purification system SPS) in a glovebox. The yields were about 93%.

While an excess of copper does not seem to be directly necessary for the reaction, the concentration of chloride anions proved to be an impacting factor the formation. Reducing the ratio of CuCl and Sb_2_S_3_ toward equimolar amounts favors the formation of CuSbS_2_. Based on the equilibrium

2AlCl_3_ + Cl^–^ ⇌ AlCl_3_ + [AlCl_4_]^−^ ⇌ [Al_2_Cl_7_]^−^

the chloride concentration must be sufficiently high to provide enough [AlCl_4_]^−^ for the precipitation of the target compound. Following these considerations, we successfully substituted one equivalent of CuCl by NaCl for the synthesis of Cu(Sb_2_S_3_)[AlCl_4_].

A direct thermal reaction of CuCl with Sb_2_S_3_ did not yield Cu(Sb_2_S_3_)Cl, but CuSbS_2_, SbCl_3_, and Cu_2_S.

### Topochemical Reaction of Cu(Sb_2_S_3_)[AlCl_4_] to Cu(Sb_2_S_3_)Cl

The water- and air-sensitive compound Cu(Sb_2_S_3_)[AlCl_4_] undergoes a topochemical reaction in contact with a diluted aqueous HCl solution. Under inert conditions, 1 mL of a 0.1 m HCl solution was carefully added to 5 mg of Cu(Sb_2_S_3_)[AlCl_4_]. Even though the reaction visually occurred immediately after contact, the crystals were kept submerged for 5 min. The crystals were washed with distilled water three times and then with dry ethanol three times, before they were dried under vacuum in a vacuum chamber.

### TEM Sample Preparation and Measurements

For the TEM/SAED/SAPEDT study, first dark particles already submerged in water were sonicated for about 6 min, producing much smaller particles, which had a red-brownish color. Then, the watered particle substance was dropped on a holey carbon film supported on a gold TEM grid, which, after drying, was mounted on a standard double-tilt TEM holder.

Conventional TEM, selected area electron diffraction (SAED), and manual selected area electron precession diffraction tomography (SA-PEDT) were performed on a FEI Tecnai F30-G2 supertwin microscope operating at 300 kV, equipped with a CCD camera (GATAN Inc.), and a standard double-tilt holder (GATAN Inc.) with a tilting range of ±46° about the holder axis and ±30° perpendicular to the holder axis. The precession electron diffraction was performed by using a DigiStar P1000 (Nanomegas). The images were stored in dm4 format, processed, and analyzed with Digital Micrograph (version 3.21.1374.0) from GATAN Inc. The Tecnai F30-G^2^ microscope was used here as a one-circle electron diffractometer.

Again, for the TEM/SAED/SAPEDT study, sonicated particles were deposited on a holey carbon film supported on a gold TEM grid. The SA-PED selected area mode was used for PED tomography. The precession angle was 1.0° or 1.57°. The SA aperture shadow on the sample grid had a diameter of about 300 nm. Three suitable Cu(Sb_2_S_3_)Cl particles were found, and thin regions of the crystallites were chosen for SA-PEDT data acquisition.

The SAPED mode was used for the precession electron diffraction tomography (SA-PEDT) experiment. Using a standard double-tilt holder (GATAN), a tilt sequence with a step width of 1° was performed manually in the range from −41° to +46° for crystal 1o, −41° to 47° for crystal 2o, and −43° to 46° for crystal 3m. The electron-beam precession angle was 1.57° for crystal 1o and 1° for crystals 2o and 3m. The collected 2D-PED data sets (88 images for 1o, 89 images for 2o, and 90 images for 3m, all in dm4 format) was converted to a TIF format data sets to be used for the reconstruction of the diffraction volume with the PETS 2.0 software package.^38^ The images were created with the VESTA 3 software.^39^

After the peak search and rotation-axis determination, the orientation matrix was obtained and the unit cell was found to be orthorhombic primitive for crystals 1o and 2o with lattice parameters for 1o: *a* = 10.36 Å, *b* = 5.69 Å, *c* = 11.38 Å (α = β = γ ≅ 90°), for 2o: *a* = 10.45 Å, *b* = 5.85 Å, *c* = 11.54 Å (α = β = γ ≅ 90°). For crystal 3m, a monoclinic primitive lattice with lattice parameter *a* = 12.04 Å, *b* = 10.45 Å, *c* = 5.88 Å, and β = 104.7° (α = γ ≅ 90°) was observed. Since the 2D electron diffraction images were not calibrated accurately for the actual microscope conditions, the unit cell parameters by the SAPEDT method are somewhat different than those obtained from X-ray powder diffraction data (see 3.4). After unit cell determination, the reflections were indexed, and the intensities were extracted and integrated and then were stored as standard *hkl* data files. A total of 1753 (938), 1760 (1064), and 1892 (1138) *hkl* reflections (no. of unique reflections in parentheses) were obtained for the crystals 1o, 2o, and 3m, respectively.

Projections (along a*, b*, and c* axes) of the integrated 3D-PED diffraction volume for o-Cu(Sb_2_S_3_)Cl (Crystal 2o) and m-Cu(Sb_2_S_3_)Cl (Crystal 3m) are shown in Figures S4 and S7, respectively. Due to stacking faults, there is diffuse electron scattering elongated along the c*_o_ (or a*_m_) axis but only in alternated Laue zones (Figures S4a and S7a), which could also be the effect of minor coherent intergrown layers.

After finding that the lamellar Cu(Sb_2_S_3_)Cl crystals consist of two intergrown phases, we intended to study the layer stacking by HRTEM on the lateral faces. Therefore, cross sections of the crystals were fabricated by using a focused Ga^+^ ion beam (FIB) microscope. Unfortunately, the subsequent TEM investigation showed that the samples were amorphisized by the FIB treatment.

### Structure Determination of o-Cu(Sb_2_S_3_)Cl

Figure S3 shows crystal 1o of Cu(Sb_2_S_3_)Cl chosen for diffraction tomography, and Figure S4 shows projections of the 3D-PED diffraction volume of crystal 2o. The *superflip* program^30^ contained in the Jana2006 software package^31^ was used for the crystal structure solution using the 3D intensity data of crystal 1o. Initially, centrosymmetric space group *Pmmm* (no. 47) was assumed, but the program indicated that the real space group is *Pmc*2_1_ (no. 26). In accordance, for crystal 2o, the statistics |*E*^2^ – 1| = 0.722 (calculated with the SHELXL program,^40^Figure S5) suggests a noncentrosymmetric structure. Changing the space group for the 1o data, the *superflip* program provided a structure solution. After analyzing the structure, taking into account the EDX analyses, interatomic distances, and atomic environments, it was concluded that the composition was Cu(Sb_2_S_3_)Cl. The preliminary refinement on *F* (31 parameters) using the Jana2006 program^31^ converged to a residual value of *R*_*g*_ = 0.269 (778 reflections with *I* > 2σ(*I*)).

After crystal structure determination and refinement of the crystals 2o and 3m, it was found that the orthorhombic and monoclinic crystals contain coherent intergrowth defects of each other phase. In the case of 1o, the intergrown is minor and does not affect much the *R*_g_ value. After introducing the second phase, the final refinement on *F*^2^ (only the second phase content was refined) resulted in 4.0% monoclinic second phase without twinning. The *R*_*g*_ value was 0.269 (33 parameters and 778 reflections with *I* > 2σ(*I*)). Similarly, for crystal 2o, the final refinement on *F*^*2*^ (33 parameters) converged to a residual value of *R*_*g*_ = 0.200 (967 reflections with *I* > 2σ(*I*)), resulting in 11% monoclinic phase content (with 4.8% and 6.5% monoclinic twin fractions). These are relatively large residual *R*_*g*_ values but fair for 3D-PED electron diffraction raw data with still some rest multibeam dynamical effect. The crystallographic data are listed in Table S1, the atomic coordinates and displacement parameters in Table S2 and selected interatomic distances are in Table 1.

### Structure Determination of m-Cu(Sb_2_S_3_)Cl

Figure S6 shows the crystal 3m of Cu(Sb_2_S_3_)Cl chosen for diffraction tomography, and Figure S7 shows projections of the 3D-PED diffraction volume of crystal 3m. The *superflip* program^30^ contained in the Jana2006 software package^31^ was also used for the crystal structure solution using the 3D intensity data of crystal 3m. Initially, the centrosymmetric space group *P*2_1_ (no. 4) was assumed, but only after some runs, the program indicated that the real space group is *P*2_1_/*m* (no. 11). After the space group was changed for the 3m data and after several runs, the *superflip* program suggested a structure solution. The preliminary refinement on *F* (31 parameters) using the Jana2006 program converged to a residual value of *R*_*g*_ = 0.380 (608 reflections with *I* > 2σ(*I*)). However, the crystal appeared to contain a (001) mirror twin, and after considering the twin (1 parameter more), the residual value dropped to *R*_*g*_ = 0.256 (32 parameters, 608 reflections with *I* > 2σ(*I*)) with a volume ratio of the twin domains of 56:44.

Analysis of the 3D-PED diffraction volume suggests that this crystal also suffers intergrowth, being mixed with the orthorhombic phase. After introducing the second phase, the final refinement on *F*^2^ (only the second phase content was refined) resulted in 35.4% orthorhombic phase content (with 44% and 20.6% monoclinic twin fractions). The *R*_*g*_ value dropped to 0.239 (33 parameters and 974 reflections with *I* > 2σ(*I*)). Again, this is a relatively large residual *R*_*g*_ value, but fair for 3D-PED electron diffraction raw data with still some rest multibeam dynamical effect. The crystallographic data are listed in Table S1, the atomic coordinates and displacement parameters in Table S3, and selected interatomic distances in Table 1.

### Powder X-ray Diffraction

X-ray powder diffraction (XRPD) patterns were measured with a Huber Image Plate Guinier Camera G670 (Huber Diffraktionstechnik GmbH & Co. KG, Rimsting, Germany), using Cu*K*_α1_ radiation, λ = 1.54059 Å. Determination of diffraction peak positions and unit cell parameters handlings were performed with the WinXPOW program.^41^ The powder was not ideal for refinement due to its strong texture. However, with the crystal structures known, a PXRD pattern (Figure S9) of the product was used to determine the lattice parameters by the Le Bail method. At room temperature, these are *a*_*o*_ = 10.617(1) Å, *b*_*o*_ = 5.898(1) Å, *c*_*o*_ = 11.730(8) Å for o-Cu(Sb_2_S_3_)Cl, and *a*_*m*_ = 12.219(8) Å, *b*_*m*_ = 10.617(1) Å, *c*_*m*_ = 5.884(1) Å, and β_m_ = 105.17(3)° for m-Cu(Sb_2_S_3_)Cl. To avoid strong correlations, *a*_*o*_ and *b*_*m*_ were constrained to be equal. The phase content in the powder was estimated to be 53(5)% o-Cu(Sb_2_S_3_)Cl and 47(5)% m-Cu(Sb_2_S_3_)Cl.

### UV–vis Spectroscopy

The absorption spectrum was recorded with a VARIAN CARY 50 UV/vis spectrometer equipped with an external diffuse reflectance accessory probe (Barrelino, Harrick Scientific). The powder sample was prepared on quartz glass placed on BaSO_4_ and measured between 300 and 1000 nm. BaSO_4_ was used as a reference for the baseline correction. The sample mass was about 10 mg. A Tauc plot, using the formula for a direct band transition, gave a bandgap of 1.54 eV.

### Quantum Mechanical Calculations

Calculations were performed within the framework of DFT as implemented in the Vienna ab initio simulation package.^33^ The plane-wave projector augmented-wave (PAW) method was used along with Perdew–Burke–Ernzerhof (PBE) exchange-correlation functional.^34^ An energy cutoff of 450 eV was set for plane-wave expansion of the calculations, and van der Waals interactions were taken into account using the many-body dispersion method.^35,36^ The Brillouin zone of the cells (28 atoms) was sampled using the Monkhorst–Pack method.^42^ The atomic structure of the system (Figure S12) was fully optimized (including the cell sizes) by using the coordinates derived from the experiments as the input. The final coordinates (for space group *P*1) are listed in Table S4. Relaxation gave rise to only small changes in the atomic coordinates, and the lateral cell size was essentially the same (10.6169/10.6175 Å before/after optimization). The transverse cell size (van der Waals gap direction) changed by about 5% (from 11.7299 to 11.1147 Å) upon optimization. We note, though, that the experimental value of the interlayer distance may be somewhat overestimated, as species intercalated between the sheets of the material may have given rise to a larger average separation between the sheets.

Electronic structure calculations at the PBE level indicated that the material has an indirect band gap of 0.8 eV. The band structure is shown in Figure 5. For comparison, we also calculated the band structure of a single sheet of o-Cu(Sb_2_S_3_)Cl. It has a slightly smaller band gap of 0.75 eV. The gap is also indirect, but the valence band maximum is shifted away from the Γ point. The total and local density of states are shown in Figure S13. As DFT with local/semilocal functionals underestimates the gap, we also carried out G0W0 calculations, which gave a quasiparticle gap of 1.6 eV, which is close to the experimental value of 1.54 eV.

## Supporting Information Available

Additional figures of the crystal structures, DSC data, electron and X-ray diffraction patterns, micrographs of the crystals used for electron diffraction, crystallographic data, UV–vis spectrum, total and local density of states, coordinates of DFT-optimized structure (PDF)

## ABBREVIATIONS

PEDT: precession electron diffraction tomography
TEM: transmission electron microscope
ED: electron diffraction
EDX: energy-dispersive X-ray
DSC: differential scanning calorimetry
SAED: selected-area electron diffraction
SA-PEDT: selected-area precession electron-diffraction tomography
DFT: density functional theory
PAW: plane-wave projector augmented-wave
PBE: Perdew–Burke–Ernzerhof
