# Supplementary material for: Electron Diffraction Tomography on Two-Phase Nanolamellae of Topochemically Synthesized Cu(Sb2S3)Cl
Source: Inorg Chem. 2024 Jun 8;63(31):14459–67. doi: 10.1021/acs.inorgchem.4c01674 (PMC11304379; doi:10.1021/acs.inorgchem.4c01674)
Supplement: Supplementary file 1 — ic4c01674_si_001.pdf [file ic4c01674_si_001.pdf]

Supporting Information

Electron Diffraction Tomography on Two-Phase Nano-Lamellae of Topochemically Synthesized $Cu(Sb_2S_3)Cl$

Wilder Carrillo-Cabrera,‡ Oliver Dreimann,† Matthias A. Grasser,† Prosun Santra,§ Silvan Kretschmer,§ Arkady V. Krasheninnikov,§ and Michael Ruck†,‡,*

† Faculty of Chemistry and Food Chemistry, Technische Universität Dresden, 01062 Dresden, Germany
‡ Max Plank Institute for Chemical Physics of Solids, 01187 Dresden, Germany
§ Helmholtz-Zentrum Dresden-Rossendorf, 01328 Dresden, German
* To whom correspondence should be addressed: michael.ruck@tu-dresden.de

Table of Contents

Crystal Structure of the Precursor $Cu(Sb_2S_3)[AlCl_4]$ S2
Synthesis S2
TEM and SA-PEDT S3
Crystal Structure Details and Crystallographic Data S5
UV-Vis Spectroscopy S9
Quantum Mechanical Calculations S10

Crystal structure of the precursor $Cu(Sb_2S_3)[AlCl_4]$

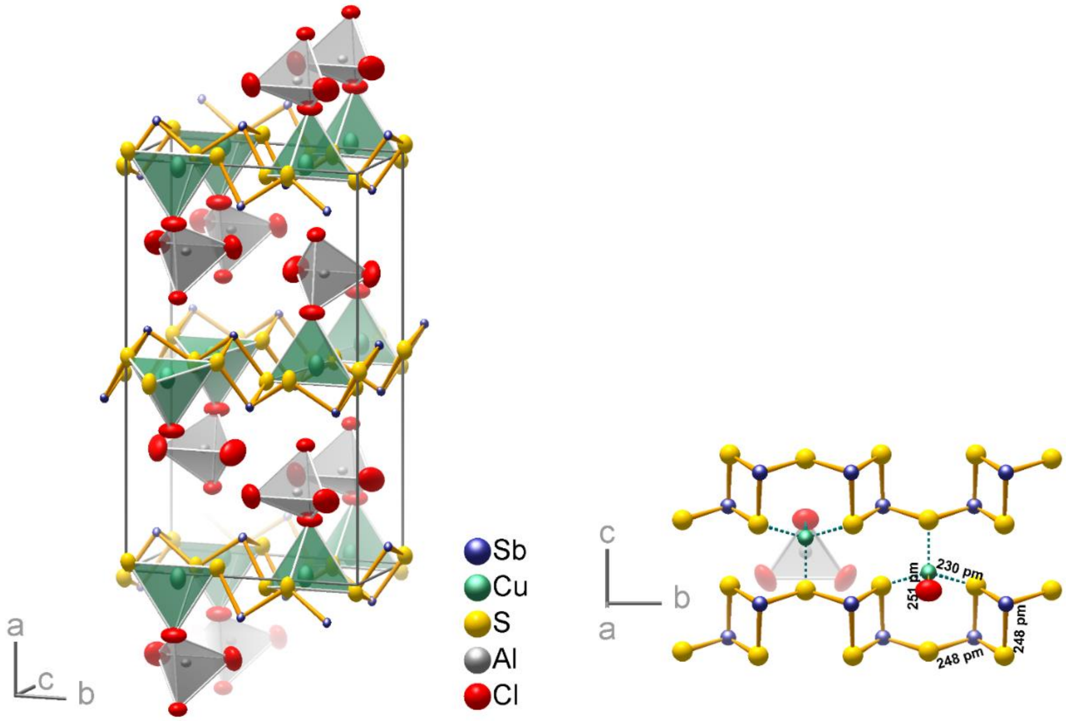

Figure S1. Unit cell of the crystal structure of $Cu(Sb_2S_3)[AlCl_4]$ (left) and top view on a single layer (right).

Synthesis

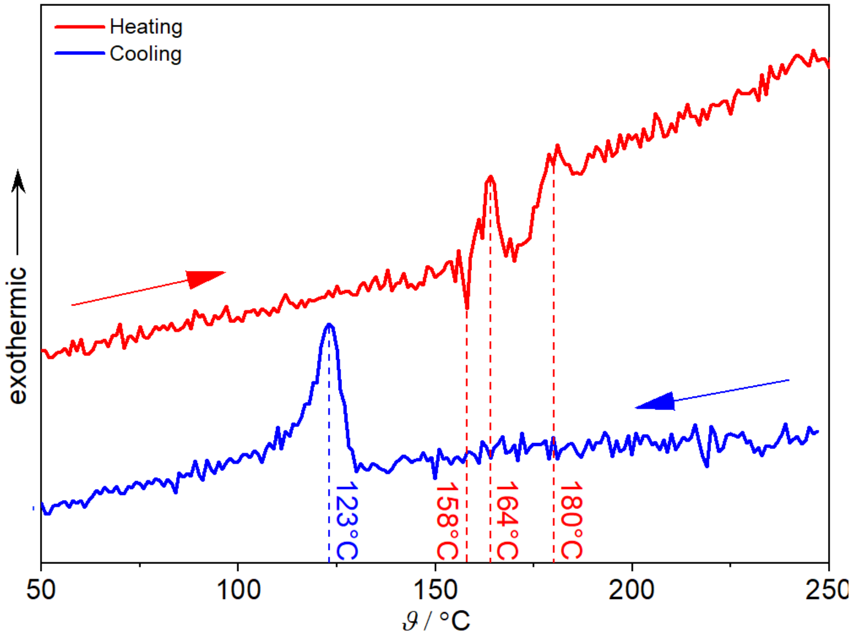

Figure S2. DSC of a 2:1 mixture of CuCl and Sb_2S_3 in the ionic liquid [BMIm]Cl·4.4$AlCl_3$.

Transmission Electron Microscopy (TEM) and Selected-Area Precession Electron Diffraction Tomography (SA-PEDT)

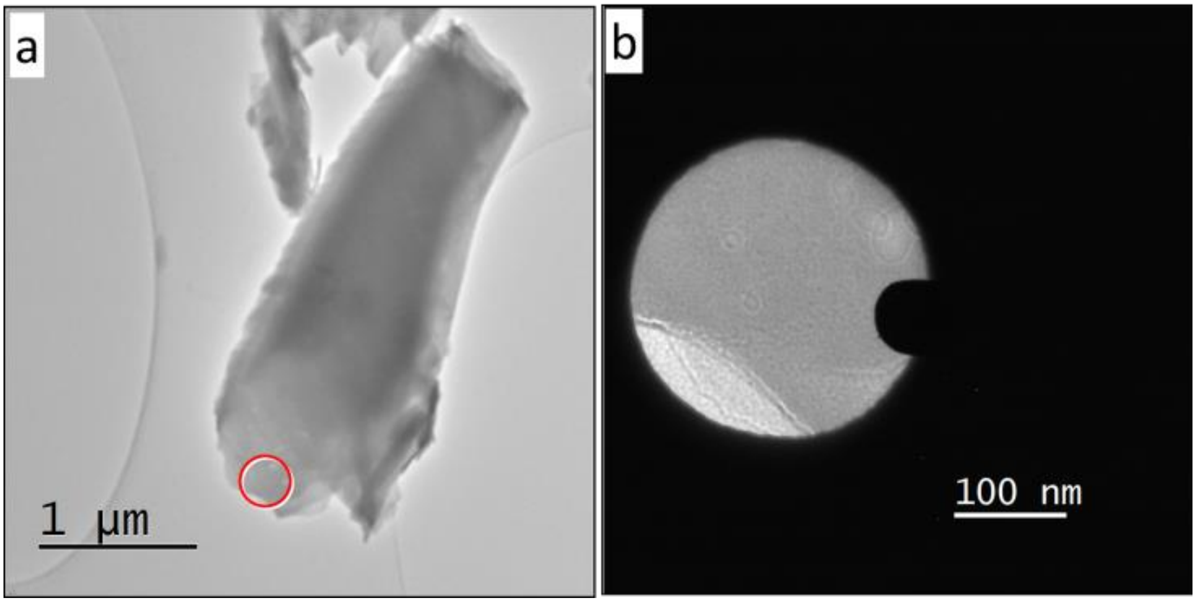

Figure S3. (a) Crystal **1o** of $Cu(Sb_2S_3)Cl$ chosen for diffraction tomography. The selected area is marked with a red circle. (b) Hole (ca. 300 nm) of the selected area (SA) aperture (ca. 300 nm) enclosing the area marked in (a) with a red circle. The top of the beam stop is also visible. The selected area of Crystal **1o** was found to consist of 90% o-$Cu(Sb_2S_3)Cl$ and 10% m-$Cu(Sb_2S_3)Cl$ (o = orthorhombic, m = monoclinic).

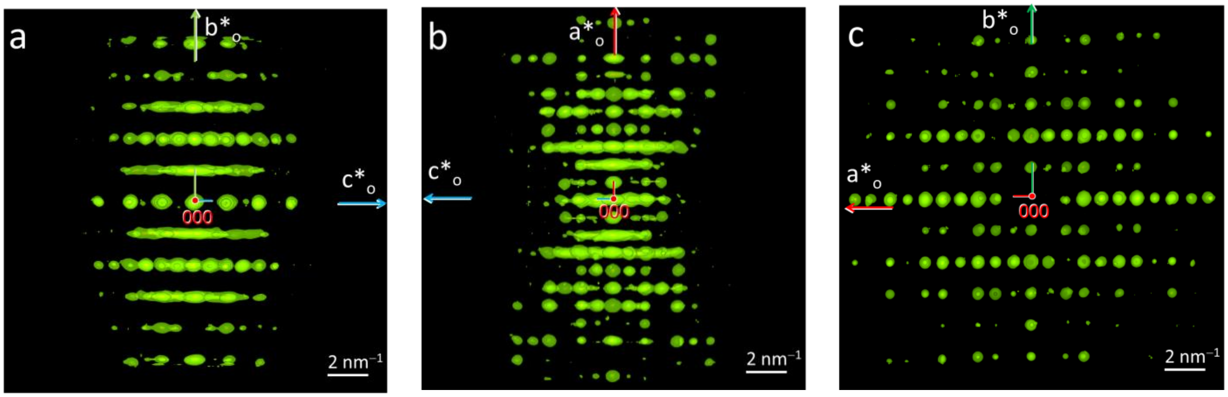

Figure S4. Projections of the 3D-PED diffraction volume of Crystal **2o**. The majority phase (84%) is orthorhombic o-$Cu(Sb_2S_3)Cl$. The projections are (a) along *a** axis, (b) along *b** axis and (c) along *c** axis. The 3D-PED diffraction volume (reciprocal-space volume) was reconstructed from 89 2D-SA-PED images (selected-area electron diffraction mode) obtained by stepwise tilt in the range –41° to 47° (tilt step 1°). The acquisition tilt axis coincides with the TEM goniometer axis (α angle tilting axis). The 3D-PED volume reconstruction was made using PETS 2.0 and the images using VESTA 3 software. Reflections in the upper Laue zones (e.g. *h*1*l*, *h*3*l*, or 2*kl*) appear somewhat elongated or diffuse, which is an effect of the superposition with the twinned monoclinic form and small domain sizes.

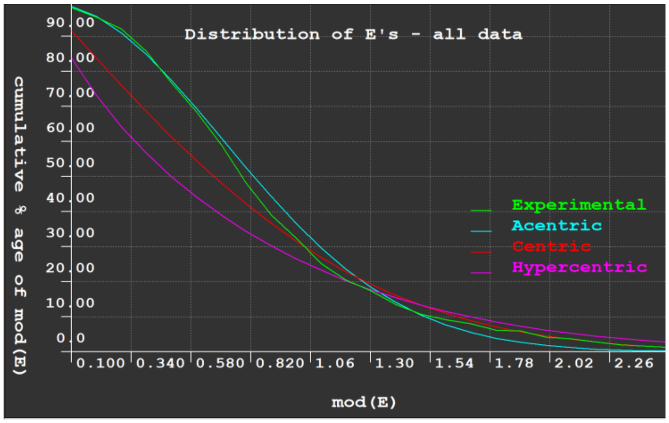

Figure S5. $|E^2 - 1|$ statistics for Crystal **2o**. The value of $|E^2 - 1| = 0.722$ suggests a non-centrosymmetric structure for o-$CuSb_2S_3Cl$. The expected value is 0.736 for an acentric structure and 0.968 for a centrosymmetric one.

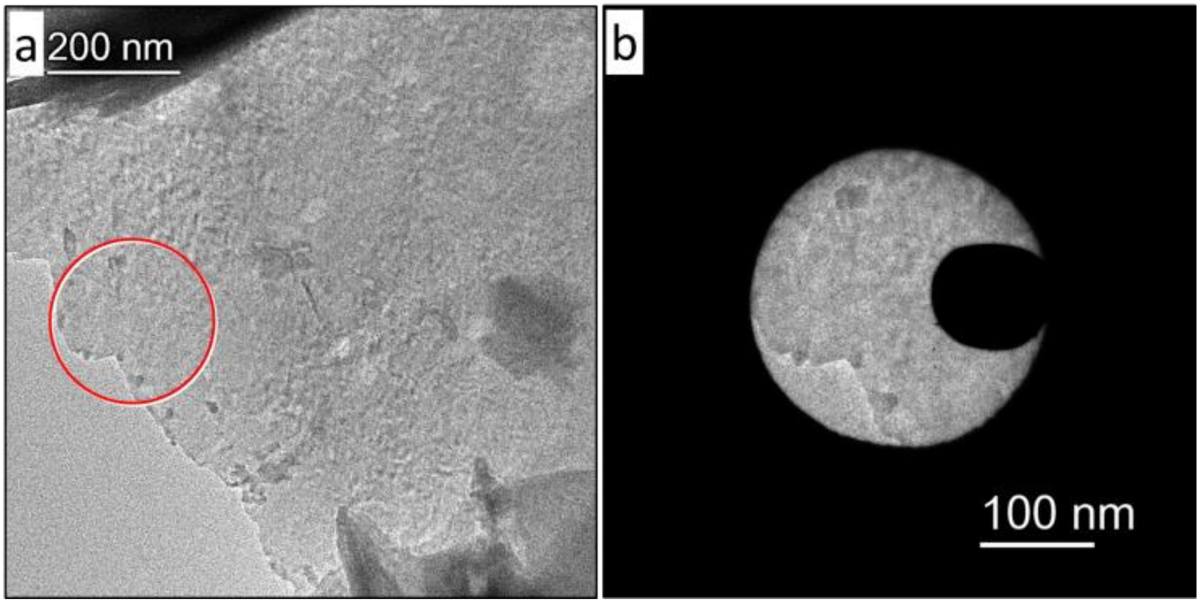

Figure S6. (a) Crystal **3m** of $Cu(Sb_2S_3)Cl$ chosen for diffraction tomography (SA-PEDT). The selected area is marked with a red circle. (b) Hole (ca. 300 nm) of the selected area (SA) aperture (ca. 300 nm) enclosing the area marked in (a) with a red circle. The top of the beam stop is also visible. After crystallographic analysis, the selected area of crystal **3m** was found to consist of 63% m-$Cu(Sb_2S_3)Cl$ and 37% o-$Cu(Sb_2S_3)Cl$.

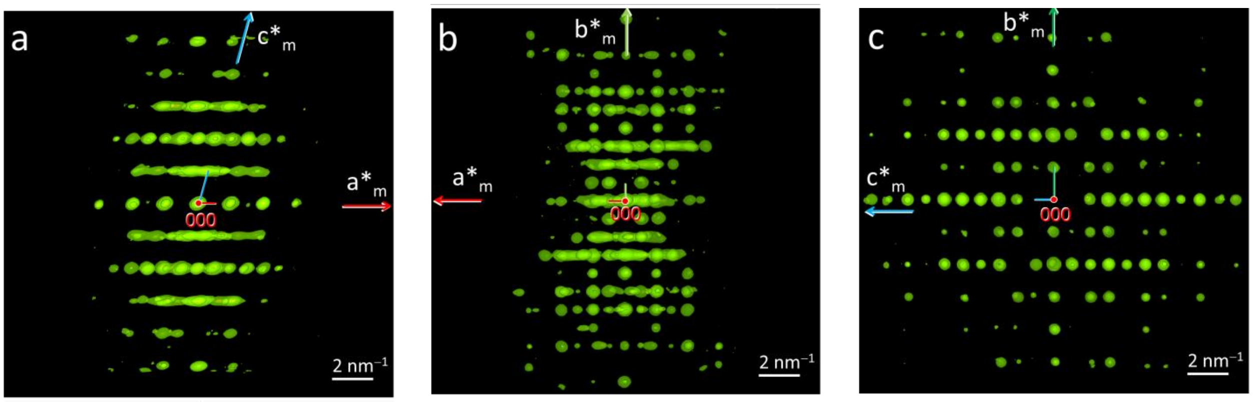

Figure S7. Projections of the 3D-PED diffraction volume of crystal **3m**. The majority phase (63%) is monoclinic m-Cu(Sb_2S_3)Cl. The projections are (a) along b^*_m axis, (b) along c^*_m axis and (c) along a^*_m axis. The 3D-PED diffraction volume (reciprocal-space volume) was reconstructed from 90 2D-SA-PED images (selected-area electron diffraction mode) obtained by stepwise tilt in the range –43° to 46° (tilt step 1°). The acquisition tilt axis coincides with the TEM goniometer axis (α angle tilting axis). The 3D-PED volume reconstruction was made using PETS 2.0 and the images using VESTA 3 software. The sequence of projections from top to bottom is correlated to that in Figure S4. Reflections in the upper Laue zones (e.g. *hk*1, *hk*3, or *h*2*l*) appear somewhat elongated or diffuse, which is an effect of the superposition of twin domains, the intergrowth with the orthorhombic form, and small domain sizes.

Crystal Structure Details and Crystallographic Data

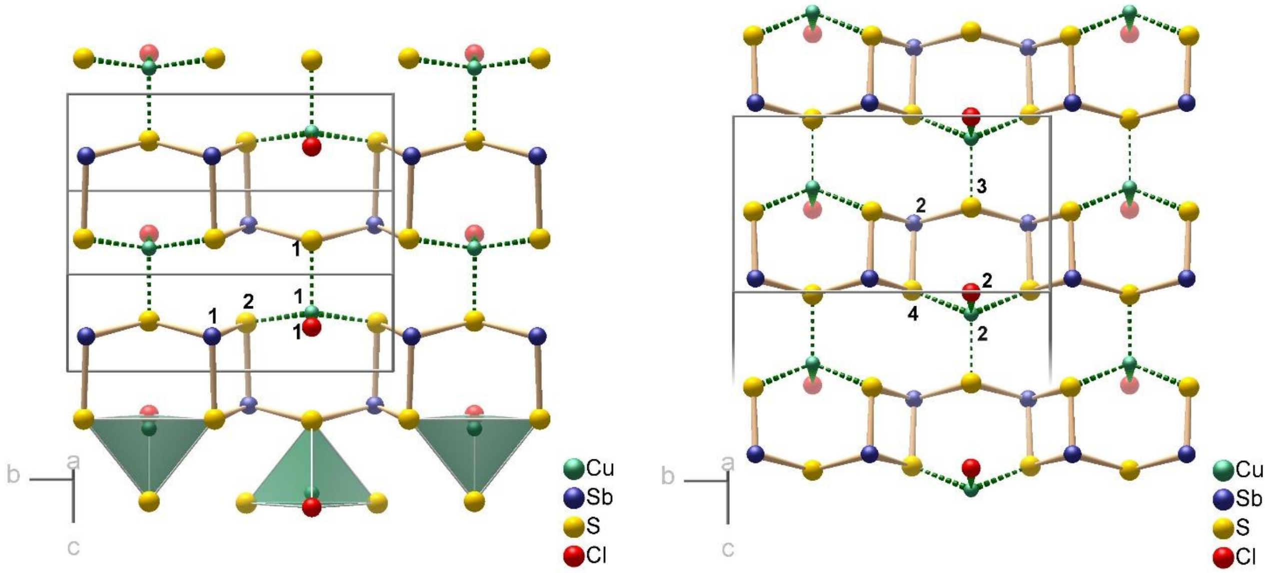

Figure S8. Top view of single $^{2}_{\infty}$[Cu(Sb_2S_3)Cl] layers around x = 0 (left) and x = 0.5 (right) in m-Cu(Sb_2S_3)Cl.

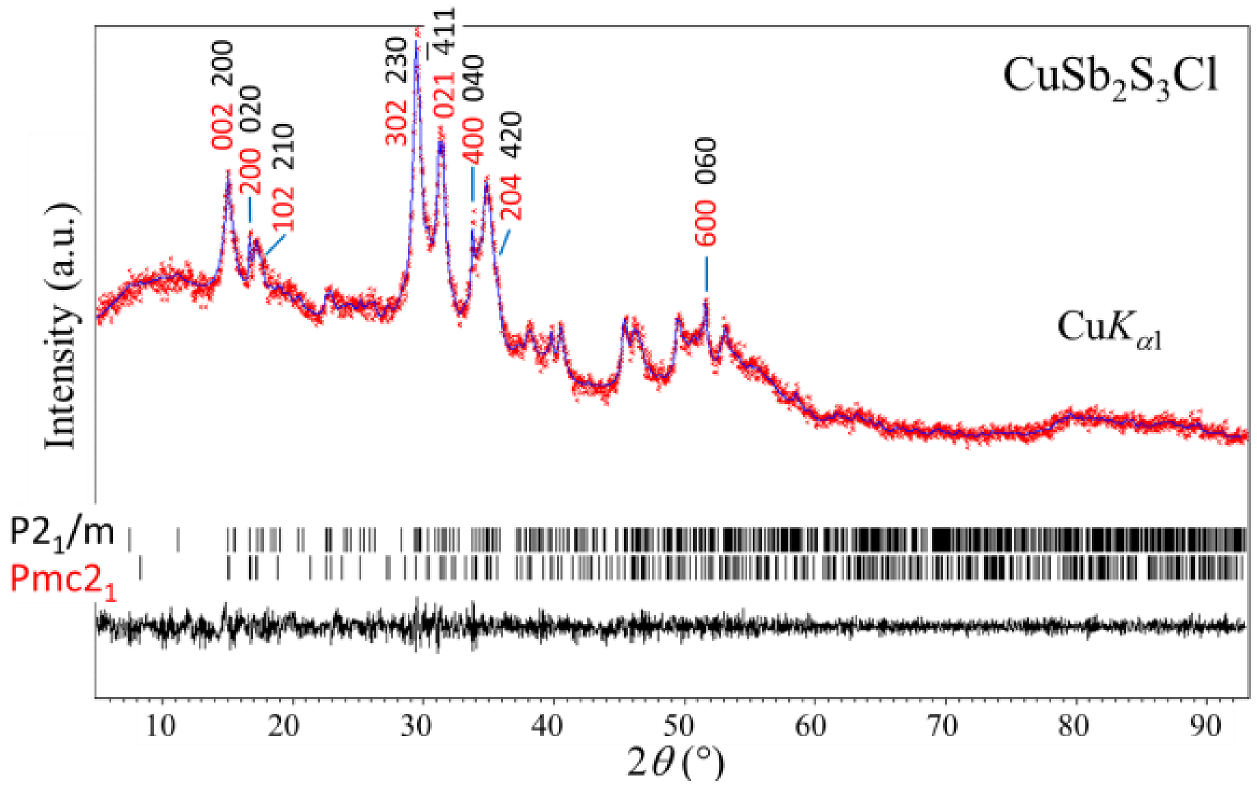

Figure S9. Powder X-ray diffraction pattern for a $Cu(Sb_2S_3)Cl$ sample ($CuK_{\alpha1}$ radiation, λ = 1.54059 Å). The Le Bail method was used to obtain lattice parameters for the orthorhombic ($Pmc2_1$) and the monoclinic ($P2_1/m$) modifications. Measured intensities are displayed as red points, the calculated pattern is displayed as a blue curve. At the bottom, reflection positions are marked by vertical black lines. The difference curve between the observed and calculated intensities is shown in black. Several reflections are indexed (red indices for o-$Cu(Sb_2S_3)Cl$ and black indices for m-$Cu(Sb_2S_3)Cl$). The powder is textured (preferred orientation of platelets), and the $h00_o/0k0_m$ reflections are significantly sharper than the other reflections.

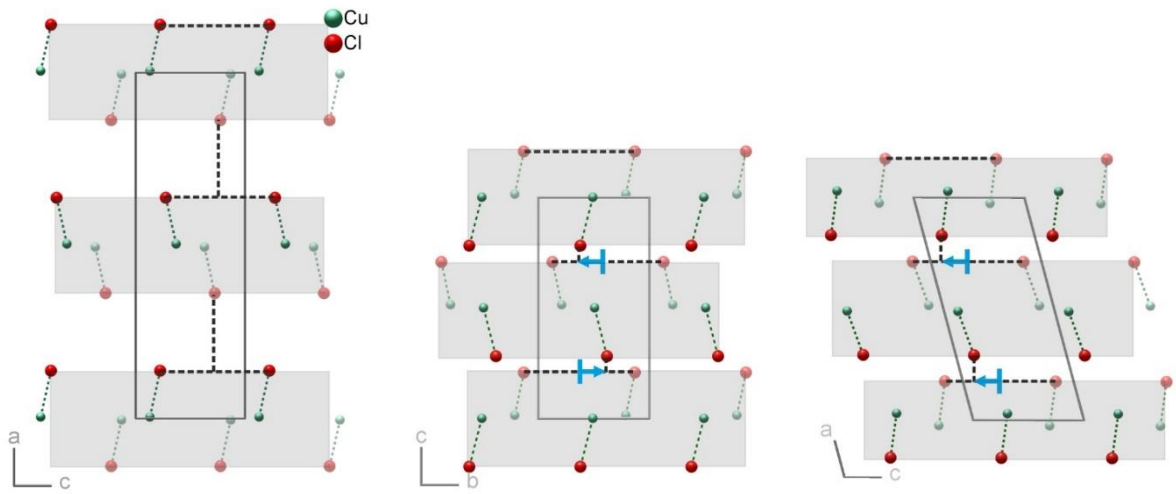

Figure S10. The stacking of the layers in $Cu(Sb_2S_3)[AlCl_4]$ and the two polymorphs of $Cu(Sb_2S_3)Cl$ can be represented in a highly reduced way, focusing on the Cu–Cl dumbbells of the $[CuS_3Cl]$ tetrahedra in the corresponding projections along the long (about 10.6 Å) axis of the layers. From left to right: Cu–Cl dumbbells of the $[CuS_3Cl]$ tetrahedra in $Cu(Sb_2S_3)[AlCl_4]$ o-$Cu(Sb_2S_3)Cl$, and m-$Cu(Sb_2S_3)Cl$. The light blue arrows indicate the layer shift in the two polymorphs of $Cu(Sb_2S_3)Cl$ relative to the precursor compound.

Table S1. Crystallographic data for $Cu(Sb_2S_3)Cl$ at 293(1) K.

Compound	o-$Cu(Sb_2S_3)Cl$		m-$Cu(Sb_2S_3)Cl$
Molar mass	438.7 g/mol		438.7 g/mol
Crystal system	orthorhombic		monoclinic
Space group	$Pmc2_1$ (no. 26)		$P\,1\,2_1/m\,1$ (no. 11)
Pearson symbol	*oP*28		*mP*28
Lattice parameters from powder X-ray diffraction	a = 10.617(1) Å<br>b = 5.898(1) Å<br>c = 11.730(8) Å<br><br>V = 734.5(5) Å^3		a = 12.219(8) Å<br>b = 10.617(1) Å<br>c = 5.884(1) Å<br>β = 105.17(3)°<br>V = 736.7(5) Å^3
Formula units	Z = 4		Z = 4
Calculated density	ρ = 3.967 g/cm^3		ρ = 3.955 g/cm^3
Data collection	Tecnai F30-G^2 super-twin electron microscope (FEI)		
Radiation	electrons, λ = 0.0197 Å		
Crystal	**1o**	**2o**	**3m**
2θ range	0.20° – 1.46°	0.20° – 1.48°	0.20° – 1.42°
Reflections, total	1753	1760	1892
Reflections, unique	938	1064	1138
Reflections, $I > 2\sigma(I)$	778	967	974
R_{int}	0.283	0.099	0.136
Absorption/extinction	uncorrected data		
Software	Jana2006[13]		
Refined parameters	32	33	33
GOF on F^2	3.63	4.90	2.67
Residual electron density	+0.75 to −0.54 e/Å^3	+0.69 to −0.53 e/Å^3	+0.68 to −0.63 e/Å^3
$R[I > 2\sigma(I)]$	0.269	0.200	0.239

Table S2. Atomic coordinates and isotropic displacements parameters for o-Cu(Sb_2S_3)Cl obtained from crystals **1o** and **2o** as well as from DFT-based structure optimization (from the space group $P1$ adapted to $Pmc2_1$).

Atom	site	x	y	z	U_{iso}/Å^2	Crystal
Cu1	2a	0	0.506(4)	0.495 (3)	0.081(7)	**1o**
		0	0.514(2)	0.502(2)	0.021(3)	**2o**
		0	0.5496	0.4495		**DFT**
Cu2	2b	1/2	0.206(3)	0.507(3)	0.066(6)	**1o**
		1/2	0.222(3)	0.502(2)	0.041(5)	**2o**
		1/2	0.2458	0.5146		**DFT**
Sb1	4c	0.3205(9)	0.6752(15)	0.3892(14)	0.064(3)	**1o**
		0.3149(6)	0.6740(12)	0.3984(13)	0.038(2)	**2o**
		0.3129	0.7045	0.3973		**DFT**
Sb2	4c	0.1870(8)	0.0314(15)	0.6116(14)	0.063(3)	**1o**
		0.1871(7)	0.0248(16)	0.6150(15)	0.048(3)	**2o**
		0.1846	0.0861	0.6104		**DFT**
S1	2a	0	0.115(4)	0.490(3)	0.056(4) [a]	**1o**
		0	0.092(3)	0.477(3)	0.019(2) [a]	**2o**
		0	0.1642	0.4874		**DFT**
S2	2b	1/2	0.607(4)	0.494(4)	0.056(4)	**1o**
		1/2	0.609(3)	0.495(2)	0.019(2)	**2o**
		1/2	0.6327	0.5206		**DFT**
S3	4c	0.1948(19)	0.606(3)	0.603(3)	0.056(4)	**1o**
		0.1942(12)	0.620(2)	0.601(2)	0.019(2)	**2o**
		0.1836	0.6632	0.5779		**DFT**
S4	4c	0.3091(16)	0.104(3)	0.444(3)	0.056(4)	**1o**
		0.3113(11)	0.112(3)	0.433(2)	0.019(2)	**2o**
		0.3167	0.1279	0.4346		**DFT**
Cl1	2a	0	0.618(4)	0.291(4)	0.060(5) [b]	**1o**
		0	0.643(3)	0.315(4)	0.047(5) [b]	**2o**
		0	0.6870	0.3024		**DFT**
Cl2	2b	1/2	0.130(4)	0.711(4)	0.060(5)	**1o**
		1/2	0.153(4)	0.721(4)	0.047(5)	**2o**
		1/2	0.1470	0.7219		**DFT**

[a] U_{iso}[S1] = U_{iso}[S2] = U_{iso}[S3] = U_{iso}[S4]. [b] U_{iso}[Cl1] = U_{iso}[Cl2] for **1o** and **2o**

Table S3. Atomic coordinates and isotropic displacements parameters for m-Cu(Sb_2S_3)Cl obtained from crystal **3m**.

Atom	site	*x*	*y*	*z*	U_{iso}/Å^2
Cu1	2*e*	0.987(4)	1/4	0.672(5)	0.046(6)
Cu2	2*e*	0.516(8)	1/4	0.868(9)	0.111(17)
Sb1	4*f*	0.1078(15)	0.9435(9)	0.867(2)	0.038(4)
Sb2	4*f*	0.3882 (14)	0.4301(8)	0.283(2)	0.040(4)
S1	2*e*	0.003(4)	1/4	0.278(5)	0.022(3) [a)]
S2	4*f*	0.078(3)	0.951(2)	0.309(4)	0.022(3)
S3	2*e*	0.506(4)	1/4	0.255(5)	0.022(3)
S4	4*f*	0.558(3)	0.937(2)	0.307(4)	0.022(3)
Cl1	2e	0.182(4)	1/4	0.855(6)	0.037(7) [b)]
Cl2	2*e*	0.717(4)	1/4	0.853(6)	0.037(7)

[a)] U_{iso}[S1] = U_{iso}[S2] = U_{iso}[S3] = U_{iso}[S4]. [b)] U_{iso}[Cl1] = U_{iso}[Cl2]

UV-Vis Spectroscopy

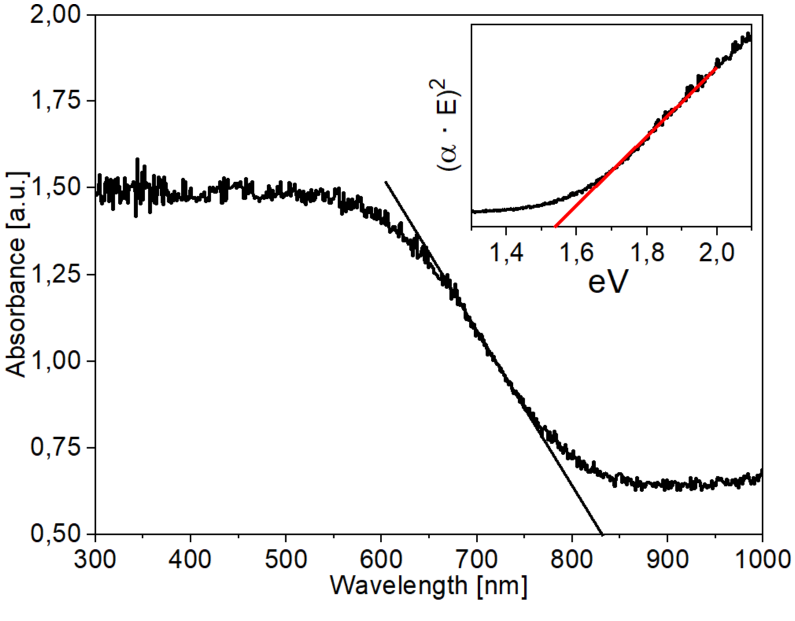

Figure S11. UV-Vis absorption spectrum of Cu(Sb_2S_3)Cl. The inlet shows a TAUC plot, estimating a direct band gap of 1.54 eV.

Quantum Mechanical Calculations

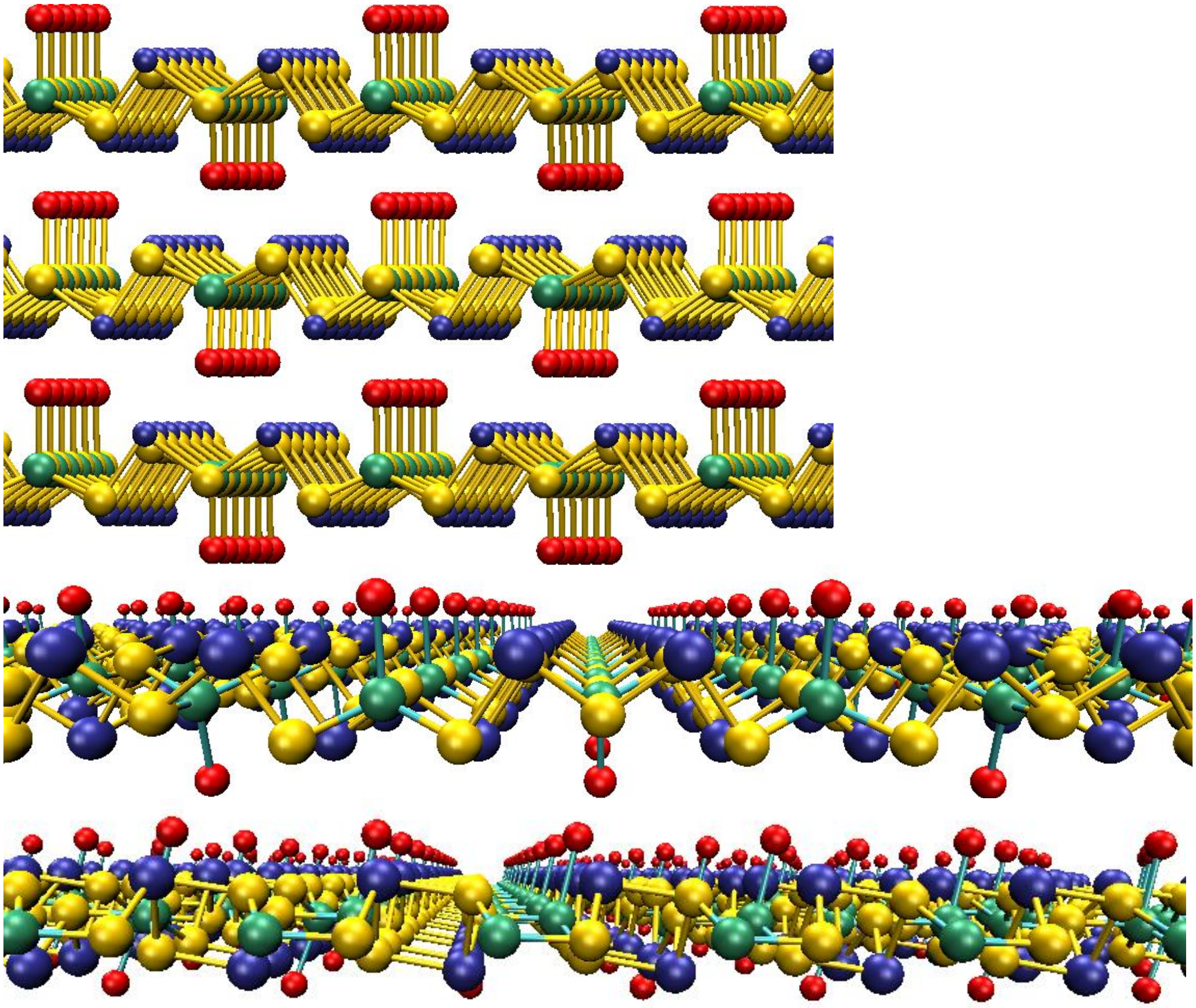

Figure S12. Crystal structure of o-Cu(Sb_2S_3)Cl and two views of a single layer after optimization. Color code for atoms: Cu green, Sb blue, Cl red, S yellow.

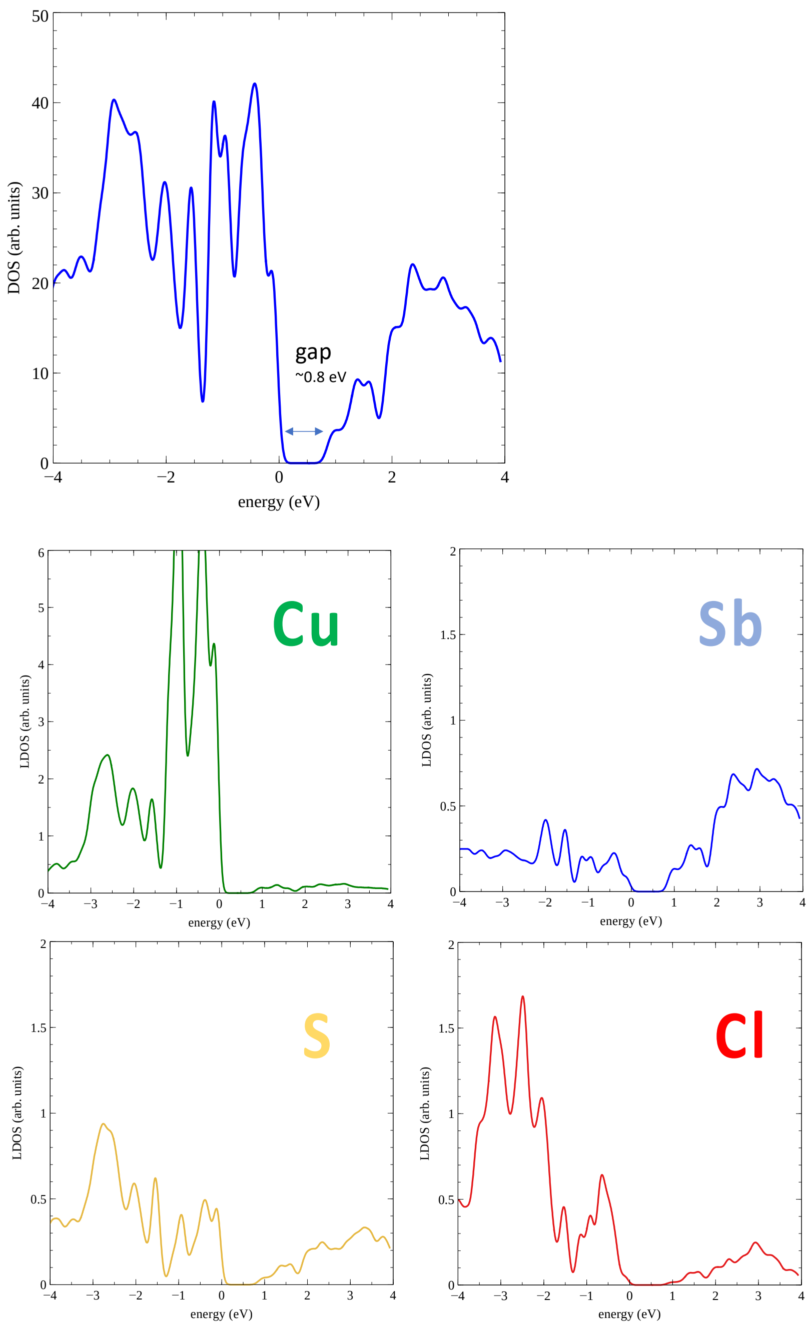

Figure S13. Total density of states (DOS) and local density of states (LDOS) for the atoms in o-Cu(Sb_2S_3)Cl (calculated with PBE functional). Note that the scale for the LDOS of Cu is different from other elements.

Table S4. Atomic coordinates for o-Cu(Sb_2S_3)Cl obtained from DFT-based structure optimization in the space group $P1$ (rounded to 5 digits). The calculated lattice parameters are a_{oc} = 10.617 Å, b_{oc} = 5.8980 Å, and c_{oc} = 11.730 Å.

Atom	x	y	z
Cu1a	0.00004	0.54956	0.44945
Cu1b	0.00061	0.44946	0.99389
Cu2a	0.50031	0.24584	0.51460
Cu2b	0.50026	0.75425	0.01411
Sb1a	0.31285	0.70448	0.39732
Sb1b	0.68615	0.29619	0.89734
Sb1c	0.31337	0.29512	0.89781
Sb1d	0.68641	0.70514	0.39750
Sb2a	0.18465	0.08614	0.61036
Sb2b	0.81453	0.91659	0.11111
Sb2c	0.18495	0.91380	0.11197
Sb2d	0.81699	0.08348	0.61126
S1a	0.00038	0.16423	0.48740
S1b	0.00032	0.83560	0.98928
S2a	0.49929	0.63274	0.52065
S2b	0.49978	0.36825	0.02100
S3a	0.18362	0.66319	0.57795
S3b	0.81594	0.34028	0.07778
S3c	0.18387	0.33623	0.07821
S3d	0.81617	0.66055	0.57768
S4a	0.31673	0.12790	0.43457
S4b	0.68407	0.87131	0.93484
S4c	0.31599	0.87091	0.93533
S4d	0.68464	0.12921	0.43586
Cl1a	0.99981	0.68701	0.30240
Cl1b	0.99848	0.31236	0.80263
Cl2a	0.49989	0.14703	0.72185
Cl2b	0.49988	0.85317	0.22173
